# *Strongyloides* Hyperinfection Syndrome Combined with Cytomegalovirus Infection

**DOI:** 10.1155/2016/1786265

**Published:** 2016-09-15

**Authors:** Fatehi Elnour Elzein, Mohammed Alsaeed, Sulafa Ballool, Ashraf Attia

**Affiliations:** ^1^Division of Infectious Diseases, Prince Sultan Military Medical City, Riyadh 11159, Saudi Arabia; ^2^Department of Nephrology, Prince Sultan Military Medical City, Riyadh 11159, Saudi Arabia

## Abstract

The mortality in* Strongyloides* hyperinfection syndrome (SHS) is alarmingly high. This is particularly common in bone marrow, renal, and other solid organ transplant (SOT) patients, where figures may reach up to 50–85%. Immunosuppressives, principally corticosteroids, are the primary triggering factor. In general, the clinical features of* Strongyloides stercoralis* hyperinfection are nonspecific; therefore, a high index of suspicion is required for early diagnosis and starting appropriate therapy. Although recurrent Gram-negative sepsis and meningitis have been previously reported, the combination of both cytomegalovirus (CMV) and strongyloidiasis had rarely been associated. We here describe a patient who survived SHS with recurrent* Escherichia coli* (*E. coli*) urosepsis and CMV infection.

## 1. Introduction

Strongyloidiasis is one of the most neglected tropical diseases. An estimated 370 million people worldwide are infected with* S. stercoralis* with the majority of the cases in tropical and subtropical countries [1]. Unlike other nematodes, these worms may persist in the human body for decades following initial infection. This longevity of* Strongyloides* is related to its unique and complex life cycle with its alternation between free-living and parasitic cycles and the propensity for autoinfection and multiplication within the infected host [2]. A review on prisoners of World War II from the United Kingdom indicated that there are still probably 300–400 veterans who remain alive in Britain and have* Strongyloides* infections [3]. Although infection is mild in immunocompetent patients, a severe and fatal disseminated disease tends to occur in immunocompromised patients. Hyperinfection syndrome develops when immunosuppression reduces the immune surveillance and results in augmentation of the normal life cycle of the parasite leading to a dramatic increase in the density of the larvae. Larvae proliferate intensely in the duodenum, migrate through the bowel wall, and then move to the lungs and back to the small bowel [4]. Immunosuppression secondary to corticosteroids is the main risk factor; however, association with human T-lymphotropic virus type I (HTLV-1) confection, organ transplant patients, or patients receiving chemotherapy are all at increased risk [5, 6]. Since the presentation of the disease is nonspecific, many patients are discovered late with an anticipated poor outcome.

## 2. Case Presentation

**Figure 1:**
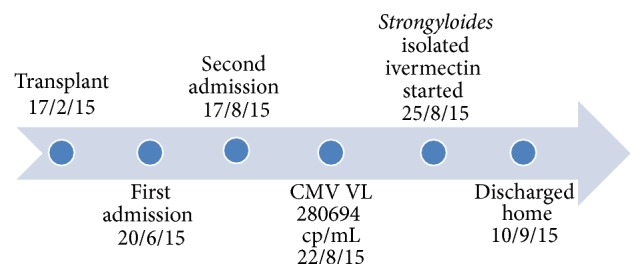
Timeline of the patient's events.

A 21-year-old student underwent a 2HLA mismatch deceased donor renal transplant on 17/2/2015 (Figure 1). He received basiliximab as induction therapy, followed by daily tacrolimus, prednisolone, and mycophenolate. On 20/6/2015 (day 0), he presented to a local hospital with nausea, vomiting, diarrhea, and 9 kg weight loss. Mycophenolate was stopped without success. Esophagogastroduodenoscopy (EGD) revealed mild reflux esophagitis, while sigmoidoscopy showed no significant changes. Rectal biopsy disclosed chronic inflammation with eosinophilic infiltrates; however, no parasites were identified. Likewise, stool analysis was negative for ova and parasites. There was no evidence of CMV colitis and CMV IgM was negative. He received albendazole 100 mg twice daily for three days with some improvement. Investigation for mycobacteria was negative including sputum smear for acid-fast bacilli (AFB), polymerase chain reaction (PCR), and culture. Chest computerized tomography (CT), abdomen, and pelvis were normal. The complete blood count was essentially normal apart from lymphopenia. The eosinophil count was also normal. Bone marrow aspiration and culture were normal. Specific stain for* Leishmania* was negative. His course in hospital was complicated by an episode of confusion. Brain CT scan disclosed old ischemic changes. Cerebrospinal fluid (CSF) examination was not performed. He was labeled as reactive psychosis and started on citalopram. He then developed fever secondary to an extended spectrum B-lactamase (ESBL) (*E. coli* ) urosepsis and bacteremia. He was treated by ertapenem for 2 weeks. A follow-up blood culture was negative. Concurrently, the renal function started to deteriorate. A renal biopsy was consistent with an early acute vascular rejection that did not respond to 3 pulses of methylprednisolone and 4 doses of antithymocyte globulin (ATG). He was reestablished on dialysis but took his own discharge on D58.

**Figure 2:**
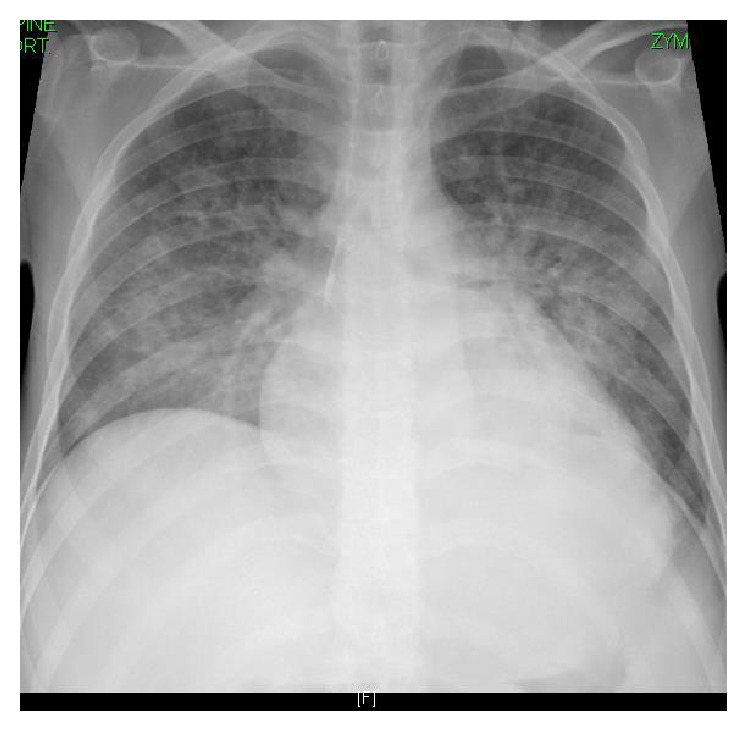
Portable CXR showing bilateral perihilar infiltrates.

**Figure 3:**
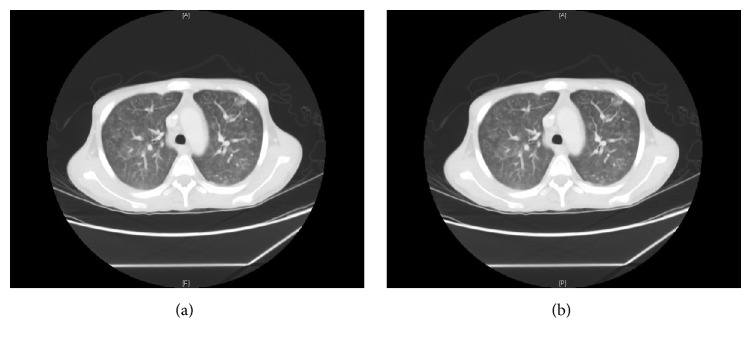
(a) CT scan showing ill-defined centrilobular nodules. (b) CT scans showing ill-defined ground glass density mainly in the upper lobes.

**Figure 4:**
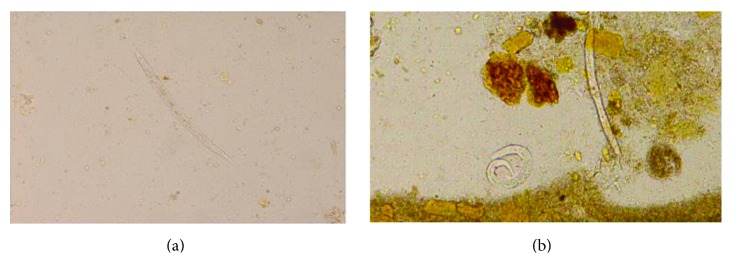
(a)* Strongyloides stercoralis* larva isolated from stool. (b) shows two* Strongyloides stercoralis* larvae in stool specimen.

He attended our Centre on D59 with similar symptoms. Diarrhea continued in the range of 8–10 times/day. His weight was 36 kg. Moreover, he complained of cough, wheezes, and shortness of breath (SOB). The oxygen saturation dropped to 88%. Portable chest X-ray (CXR) showed bilateral perihilar infiltrates (Figure 2), while CT scan revealed bilateral widespread centrilobular nodules of ground glass opacity consistent with hypersensitivity pneumonitis or bronchiolitis (Figures 3(a) and 3(b)). Echocardiogram disclosed a normal left ventricular systolic function with an ejection fraction of 55%. Additionally, there was no clinical or radiological improvement despite repeated ultrafiltration sessions. Oseltamivir, piperacillin/tazobactam, bronchodilators, and supplemental oxygen were started. Bronchoscopy and bronchoalveolar lavage (BAL) were negative for MERS-CoV, influenza viruses,* Pneumocystis jiroveci*, and parasites. Complete blood count showed hemoglobin of 8.2 g/dL, WBC of 15.4 10^9^ cells/L, and eosinophil of 0.5 10^9^ cells/L. The serum ferritin and C-reactive protein were high at 1947 ng/mL and 279 mg/L successively (normal range: ferritin of 30–400 ng/mL and CRP of 0–6 mg/L). The serum albumin was 27 g/L. Both blood and urine cultures were positive for multidrug-resistant (MDR)* E. coli* for which he received 2 weeks of meropenem. Stool examination on D66 showed numerous* S. stercoralis* larvae on multiple occasions (Figures 4(a) and 4(b)). Nevertheless, repeated sputum analysis and BAL specimen were negative for parasites. A diagnosis of recurrent* E. coli* bacteremia and hyperinfection syndrome was made. Immunosuppressant drugs were discontinued, and steroids were tapered. He was started on oral ivermectin 200 *µ*g/kg once daily for two weeks. The stools turned negative by the third day of treatment. Interestingly, CMV DNA viral load was also elevated to 280694 cp/mL on D64, 3 days before starting ivermectin treatment, and regressed to <259 cp/mL after 3 weeks of intravenous ganciclovir. Prior to discharge, his weight increased by 11 kg, pulmonary infiltrates regressed on CXR, and oxygen saturation remained 99% on room air. He was discharged home on D83 on valganciclovir, cotrimoxazole, and prednisolone. Follow-up on day 158 showed increasing body weight to 73 kg, hemoglobin of 14.6 g/dL, WBC of 7.8 10^9^ cells/L, eosinophil of 0.1 10^9^ cells/L, and normal albumin. He is now been considered for a second transplant.

## 3. Discussion

In addition to reactivation in chronically infected recipients, SOT recipients may acquire this infection through infected graft or acquisition of a new infection. It is challenging to identify our patient's source of infection. In general, strongyloidiasis is uncommon in Saudi Arabia; however, a number of organ donors were from the Indian subcontinent and Southeast Asia, where the infection is more prevalent. Consequently, graft-related infection is being increasingly reported from the Middle East [7, 8]. The donor is of Indian origin but his eosinophil count was normal and the twin recipient of the other kidney was not affected. It is worth noting that the other recipient was not on cyclosporine. Three Kuwaiti kidney transplant recipients, from two deceased donors, died within 2 months from hyperinfection with* Strongyloides* [9]. The fourth recipient of the twin kidney was on cyclosporine and did not manifest a disease. There is increasing evidence that cyclosporine has direct antiparasitic activity, and it may provide protection against hyperinfection syndrome [8, 10, 11]. HTLV-1 infection is a well-known risk factor for hyperinfection syndrome. The high production of IFN-*γ* observed in patients coinfected with HTLV-1 and* S. stercoralis* decreases the production of IL-4, IL-5, IL-13, and IgE, molecules that participate in the host defence mechanism against helminthes. Moreover, there is a decrease in the efficacy of treatment of* S. stercoralis* in patients coinfected with HTLV-1 [12]. Conversely, combined CMV and strongyloidiasis had been reported in only a few cases and customarily detected at postmortem [13]. CMV targets different subsets of antigen-presenting cells leading to short-lived immunosuppression in immunocompetent as well as immunosuppressed patients [14].

This patient had extensive pulmonary infiltrates with ground glass appearance and wide centrilobular nodules. The differential diagnosis at the time included pulmonary edema and ARDS; however, aggressive fluid removal did not lead to either clinical or radiological improvement. Although CMV was not isolated in the BAL analysis, CMV pneumonitis remains a likely diagnosis. In a Korean study of CMV pneumonia in non-AIDS immunocompromised patients, the most common finding in HRCT scans included bilateral mixed areas of ground glass opacity, poorly defined centrilobular small nodules, and consolidation [15]. Both hypersensitivity and superimposed bacterial infection could have contributed to the radiological changes that regressed prior to discharge.

Generally, hyperinfection syndrome is associated with significant morbidity and mortality that can be avoided by early diagnosis and treatment. If untreated, the mortality rate of disseminated disease approaches 100%. Hematopoietic stem cell transplant recipients have particularly poor outcomes, with mortality reaching up to 85%. Equally, mortality can reach up to 50% in renal transplant [4]. This is partly related to a delay in the diagnosis and initiation of treatment, as well as the accompanying Gram-negative sepsis. It is proposed that larvae carry colonic bacteria during their migration to the venous system leading to translocation into the blood and other tissues including the meninges. Commonly reported organisms include Gram-negative rods such as* E*.* coli* and Gram-positive cocci, for instance,* Streptococcus bovis*. Recurrent* E. coli* bacteremia and* S. bovis* meningitis are one manifestation of this translocation [16, 17]. Thus,* S. bovis* bacteremia and/or meningitis should prompt a search for* Strongyloides* infection in transplant patients.

The diagnosis of hyperinfection syndrome can be difficult. Although eosinophilia is a common finding in patients with chronic* Strongyloides* infection, it is a very unreliable predictor of hyperinfection syndrome. Up to 75% of people with chronic strongyloidiasis have mild peripheral eosinophilia or elevated IgE levels. However, peripheral eosinophilia was present in only 12/73 cases (16.4%) of disseminated infection [18, 19]. Similar to our patient's presentation, the absence of eosinophilia while receiving corticosteroid therapy cannot reliably exclude underlying* Strongyloides* infection. The laboratory confirmation of strongyloidiasis is based mainly on the detection of* Strongyloides* larvae by microscopic examination of the stools, sputum, or CSF in disseminated infection. The initial direct fecal smear stool examination in the referring hospital was negative, while multiple specimens were positive in our hospital. This could be due to the early presentation in the first admission before the florid syndrome at the time he presented to our hospital, as well as the poor technique for parasite isolation. A single stool examination fails to identify larvae in up to 70% of cases; the diagnostic sensitivity for* S. stercoralis* rises to 60–70% with 3 or more stool samples, while up to seven stool exams are required to reach sensitivity of 100% [20]. Stool agar plate culture (APC) or Baermann culture techniques had higher yield when compared to microscopy using the Kato-Katz technique [21]. A recent meta-analysis on the evaluation of conventional parasitological methods found the top sensitivity (89%) for APC, followed by the Baermann technique (72%), FECT (48%), and direct wet smear (21%) [22]. Different molecular methods were found to be more sensitive and reliable in detecting* S. stercoralis*. The sensitivity and specificity of nested PCR were 100 and 91%, respectively, while a real-time PCR yielded 100% sensitivity and 91.6% specificity [23].

An early diagnosis in this patient could have been secured in the presence of serology. Serological methods are the most sensitive available diagnostic tools. A variety of antigens have been used to develop serological tests. ELISA test had a negative predictive value of 98% and is an excellent screening test for strongyloidiasis [24]. A number of studies demonstrated high sensitivity with specificity of >90% in most of reports. These tests can be used to make the diagnosis and screening and more importantly as a possible test of cure [22]. This underscores the importance of maintaining serological tests in all transplant centers in order to avoid this serious infection.

Treatment options include albendazole, thiabendazole, and ivermectin. In a recent Cochrane review, ivermectin has been proven to be more effective than albendazole (RR: 1.79; CI: 1.55 to 2.08) and equally effective to thiabendazole but more tolerable than it (RR: 1.07; CI: 0.96 to 1.20) [25]. It is usually given orally; subcutaneous injection (veterinary formulation) and retention enema were used, too. The duration of treatment of hyperinfection syndrome is variable. The CDC recommends ivermectin, 200 *µ*g/kg per day orally, until stool and/or sputum exams are negative for 2 weeks. If possible, immunosuppressive therapy should be stopped or reduced.

To the best of our knowledge, this is the first case report of* Strongyloides* hyperinfection with concurrent (CMV) infection and Gram-negative sepsis in a Saudi patient. Despite a very aggressive disease, this patient had a favorable outcome and is now evaluated for a second transplant.
